# Regulated N-Terminal Modification of Proteins Synthesized Using a Reconstituted Cell-Free Protein Synthesis System

**DOI:** 10.1021/acssynbio.3c00191

**Published:** 2023-06-16

**Authors:** Rena Matsumoto, Tatsuya Niwa, Yasuhiro Shimane, Yutetsu Kuruma, Hideki Taguchi, Takashi Kanamori

**Affiliations:** † GeneFrontier Corporation, 273-1 Kashiwa, Kashiwa, Chiba 277-0005, Japan; ‡ Cell Biology Center, Institute of Innovative Research, Tokyo Institute of Technology, Yokohama 226-8503, Japan; § Institute for Extra-cutting-edge Science and Technology Avant-garde Research (X-star), Japan Agency for Marine-Earth Science and Technology (JAMSTEC), 2-15 Natsushima-cho, Yokosuka, Kanagawa 237-0061, Japan

**Keywords:** cell-free protein synthesis, PURE system, N-terminal modification, acetylation, myristoylation, giant vesicles

## Abstract

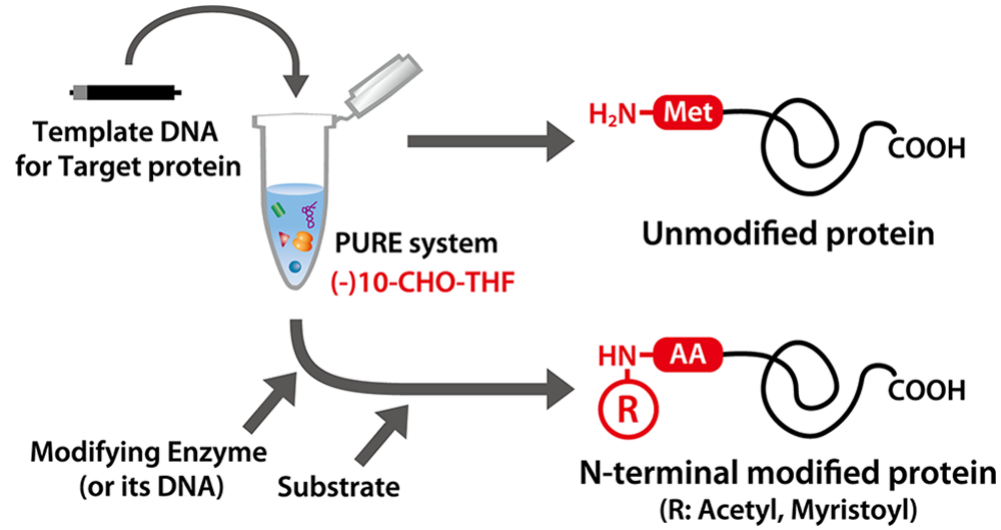

The N-terminal modification of nascent proteins, such as acetylation and myristoylation, is one of the most abundant post-translational modifications. To analyze the function of the modification, it is important to compare the modified and unmodified proteins under defined conditions. However, it is technically difficult to prepare unmodified proteins because cell-based systems contain endogenous modification systems. In this study, we developed a cell-free method to conduct N-terminal acetylation and myristoylation of nascent proteins *in vitro* using a reconstituted cell-free protein synthesis system (PURE system). Proteins synthesized using the PURE system were successfully acetylated or myristoylated in a single-cell-free mixture in the presence of modifying enzymes. Furthermore, we performed protein myristoylation in giant vesicles, which resulted in their partial localization to the membrane. Our PURE-system-based strategy is useful for the controlled synthesis of post-translationally modified proteins.

## Introduction

The N-terminal modification of nascent proteins, including acetylation and myristoylation, is one of the most abundant post-translational modifications, especially in eukaryotes.^1,2^ N-terminal acetylation is mediated by N-acetyltransferase (NAT). NatA and NatB are known as major NATs among those reported to date.^3−5^ NatB catalyzes the transfer of the acetyl residue from acetyl-coenzyme A (acetyl-CoA) to the α-amino residue of the initial methionine of the nascent protein. Conversely, NatA acetylates the α-amino residue of the second amino acid after the removal of the initial methionine by methionine aminopeptidase (MAP).^3−5^ N-Myristoyltransferase (NMT) catalyzes the transfer of the myristoyl residue from myristoyl-CoA to the second glycine after the removal of the initial methionine.^6,7^ Acetylation is required for many processes in cells, including protein–protein interaction, subcellular localization, and degradation,^2−5^ while myristoylation is crucial for the localization of the target protein to the membrane.^6,7^ To examine the function of N-terminal-modified proteins *in vitro*, modified and unmodified proteins have to be prepared and compared under defined conditions. However, it is difficult to prepare unmodified recombinant proteins because most cells, including *Escherichia coli*, endogenously contain N-terminal-modifying enzymes.

The PURE system is a reconstituted cell-free protein synthesis system based on the *E. coli* translation system.^8^ It contains only the purified ribosome, translation factors, tRNA, and substrates and does not contain any other molecules such as nucleases, proteases, and other metabolic systems. Therefore, proteins synthesized in the PURE system cannot be modified because no N-terminal-modifying enzymes are present. One advantage of the PURE system is that it facilitates the effortless arrangement of reaction conditions required for target protein synthesis. For example, our group previously reported that aglycosylated full-length immunoglobulin G could be synthesized by adjusting the redox conditions in the reaction mixture and supplying molecular chaperones.^9^

In this study, we performed *in vitro* acetylation or myristoylation at the N-terminus of a protein synthesized within the PURE system. As a result, we succeeded in synthesizing N-terminal-modified proteins by synthesizing modifying enzymes simultaneously or adding them after synthesis. These results demonstrate the ease and speed of the preparation of both unmodified and N-terminally modified proteins in a completely regulated manner. By use of our developed methods, the function of N-terminal modification can be easily studied. Indeed, we performed protein myristoylation in giant vesicles, which resulted in their partial localization to the membrane. Our results show that the PURE system is useful for the preparation of unmodified as well as post-translationally modified proteins.

## Results and Discussion

### Protein Synthesis without 10-CHO-THF

**Figure 1:**
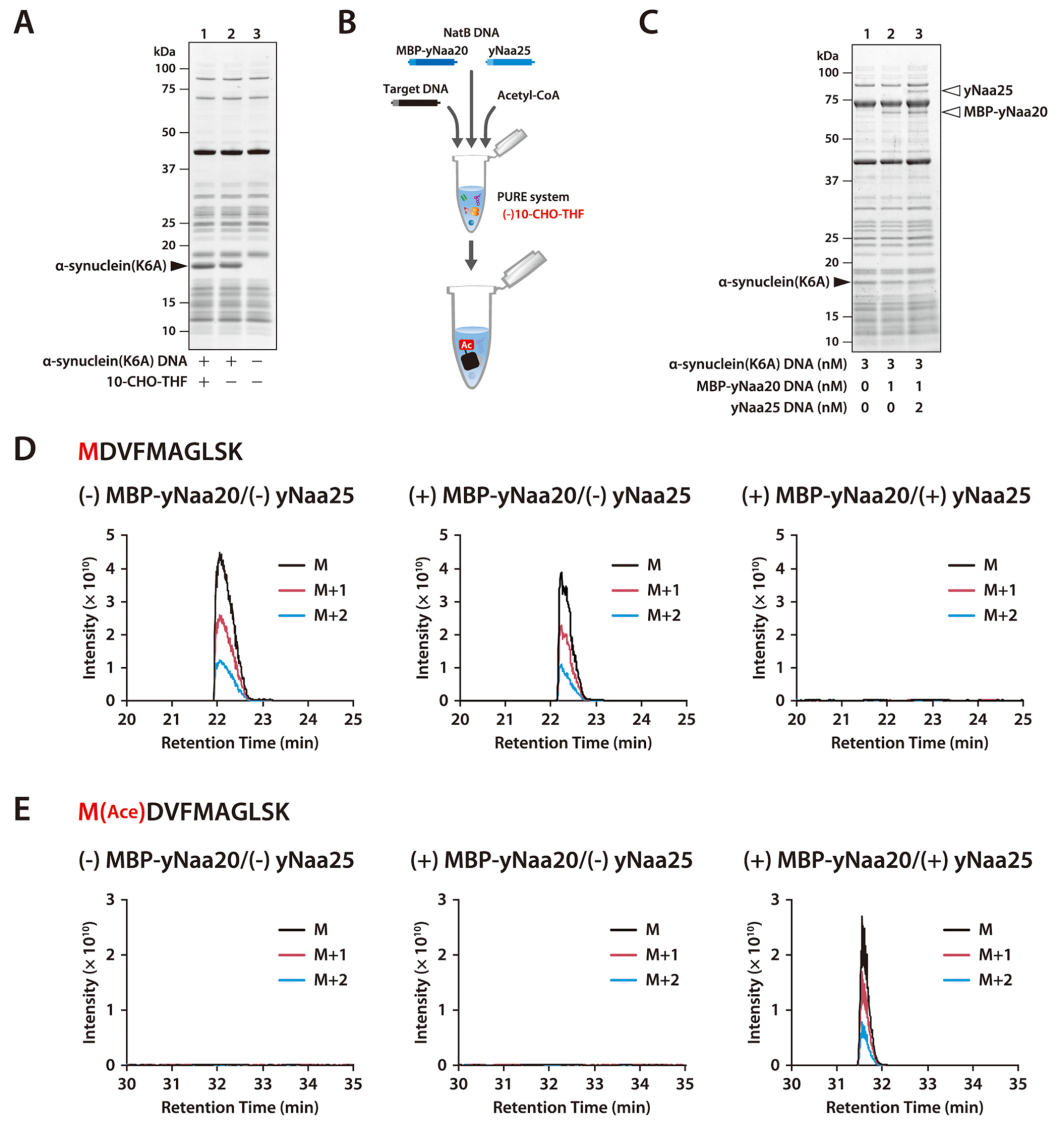
NatB-mediated N-terminal acetylation of α-synuclein(K6A). (A) SDS-PAGE analysis of α-synuclein(K6A) synthesized in the presence or absence of 10-formyl tetrahydrofolate (10-CHO-THF) at 37 °C for 4 h. (B) Schematic outline of NatB-mediated N-terminal acetylation. (C) SDS-PAGE analysis of α-synuclein(K6A) cosynthesized with MBP-yNaa20 and yNaa25. α-Synuclein(K6A) DNA (3 nM), MBP-yNaa20 DNA (1 nM), and yNaa25 DNA (2 nM) were added to the PURE system ((−) 10-CHO-THF) containing 5 μM DnaK, 1 μM DnaJ, 1 μM GrpE, and 1 mM acetyl-CoA, as indicated. The reaction mixture was incubated at 23 °C for 24 h. (D) Extracted ion chromatograms of the unmodified N-terminal peptide derived from α-synuclein(K6A) after trypsin digestion (MDVFMAGLSK, M = 549.7697 Da, M+1 = 550.2712 Da, M+2 = 550.7710 Da, *z* = 2). The retention time was confirmed by the peptide search results from the MS/MS spectral search. (E) Extracted ion chromatograms of the acetylated N-terminal peptide derived from α-synuclein(K6A) after trypsin digestion (M[Ace]-DVFMAGLSK, M = 570.7750 Da, M+1 = 571.2765 Da, M+2 = 571.7764 Da, *z* = 2). The retention time was confirmed by the peptide search results from the MS/MS spectral search.

Formylmethionine is used as the initial amino acid of the nascent protein in *E. coli*, and the PURE system contains a formyl donor, 10-formyl tetrahydrofolate (10-CHO-THF).^8^ However, the α-amino residue of the initial methionine cannot be formylated for N-terminal modification. First, we investigated whether target proteins used in this study could be efficiently synthesized using the PURE system without 10-CHO-THF. We used α-synuclein(K6A), CPR1(10aa)(Q3E)-sfGFP, and Goα(8aa)(C3S)-sfGFP as model proteins for NatB-mediated acetylation, NatA-mediated acetylation, and NMT-mediated myristoylation, respectively (Supplementary Figure 1 and Supplementary Table 1). α-Synuclein(K6A) is a mutant α-synuclein in which the sixth lysine is replaced with alanine.^10^ We analyzed the N-terminal-modified peptides by mass spectrometry (MS) analysis after digestion by trypsin, which digests the peptide bonds after lysine and arginine. Because short peptides are difficult to analyze by MS, we used mutant α-synuclein. CPR1(10aa)(Q3E)-sfGFP and Goα(8aa)(C3S)-sfGFP are fusion proteins consisting of the N-terminal 10 amino acids of yeast CPR1^11^ and the N-terminal 8 amino acids of the human Goα subunit^12^ fused to sfGFP, respectively. To facilitate detection through mass spectrometry, glutamine at the third position in CPR1(10aa)(Q3E)-sfGFP and cysteine at the third position in Goα(8aa)(C3S)-sfGFP were substituted with glutamic acid and serine, respectively. Synthesis of the target protein α-synuclein(K6A) using the PURE system without 10-CHO-THF resulted in almost the same amount of protein as in the synthesis of α-synuclein(K6A) with 10-CHO-THF (Figure 1A and Supplementary Figure 2C). The synthesis of other proteins using the PURE system without 10-CHO-THF was also tested, but the synthesis efficiency was dependent on the individual protein (Supplementary Figure 2), *e.g.*, CPR1(10aa)(Q3E)-sfGFP was reduced by approximately 20%, whereas Goα(8aa)(C3S)-sfGFP was reduced by approximately 60%. These results indicate that 10-CHO-THF is not essential for protein synthesis within the PURE system but affects the efficiency of protein synthesis depending on the properties of the protein, especially its N-terminal sequence.

### NatB-Mediated Acetylation of the Initial Methionine of the Synthesized Protein

Yeast NatB is a heterodimer consisting of yNaa20 (encoded by NAT3) and yNaa25 (encoded by MDM20).^13^ yNaa20 has transferase activity, and yNaa25 is an auxiliary protein. When yNaa20 and yNaa25 were synthesized in the PURE system supplemented with molecular chaperone proteins (DnaK, DnaJ, and GrpE) at 37, 30, or 23 °C, synthesized yNaa25 was soluble at 30 and 23 °C, but synthesized yNaa20 was insoluble even at 23 °C (Supplementary Figure 3A). Therefore, we attempted to improve the solubility of yNaa20 by fusing it with maltose-binding protein (MBP), a soluble protein. MBP-yNaa20 was synthesized as a soluble protein at all tested temperatures (Supplementary Figure 3B). Since yNaa20p and yNaa25p form heterodimers, we simultaneously synthesized both proteins in the same tube. When two or more proteins are synthesized simultaneously in the same PURE system reaction mixture, the amount of synthesized proteins can be controlled by regulating the input amount of each template DNA.^9,14^ In the case of NatB, a 1:2 or 1:4 ratio of the template DNA of MBP-yNaa20 and yNaa25, respectively, was used to achieve the equimolar products at 23 °C (Supplementary Figure 3C,D). When α-synuclein(K6A) was synthesized with or without NatB in the presence of acetyl-CoA (Figure 1B), the mobility change of synthesized α-synuclein(K6A) between reactions with or without NatB could not be detected *via* SDS-PAGE (Figure 1C). Therefore, the N-terminal peptide of the α-synuclein(K6A) was analyzed through MS. The signals derived from the precursor ions of the non-acetylated N-terminal peptide (MDVFMAGLSK) disappeared, and those corresponding to the acetylated N-terminal peptide (M[Ace]-DVFMAGLSK) appeared in the presence of both MBP-yNaa20 and-yNaa25 (Figure 1D,E and Supplementary Figure 4A,B). We confirmed that the MS/MS spectra corresponding to the modified peptides were annotated by the search engine at the same retention times as those of the peaks (Supplementary Figure 4C). Yeast NatB associates with ribosomes and acetylates nascent proteins in a cotranslational manner.^15^ However, this result indicates that yeast NatB can also react with the nascent protein synthesized by the *E. coli* ribosome and that both yNaa20 and yNaa25 are necessary for the acetylation reaction.^13^

### NatA-Mediated Acetylation after Removal of N-Terminal Methionine

**Figure 2:**
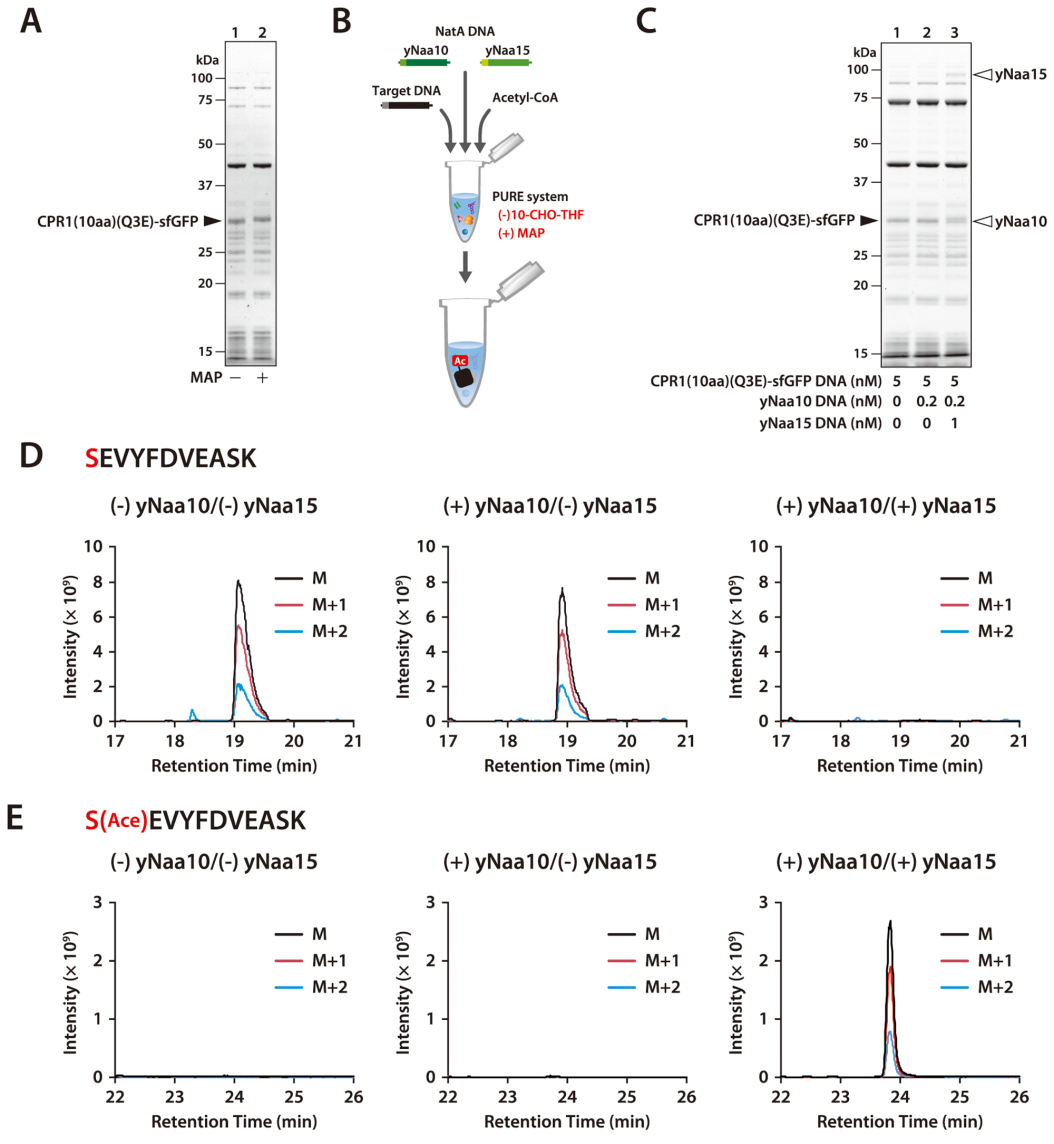
NatA-mediated N-terminal acetylation of CPR1(10aa)(Q3E)-sfGFP. (A) SDS-PAGE analysis of CPR1(10aa)(Q3E)-sfGFP synthesized in the presence or absence of methionine aminopeptidase (MAP). (B) Schematic outline of NatA-mediated N-terminal acetylation. (C) SDS-PAGE analysis of CPR1(10aa)(Q3E)-sfGFP cosynthesized with yNaa10 and yNaa15. CPR1(10aa)(Q3E)-sfGFP DNA (5 nM), yNaa10 DNA (0.2 nM), and yNaa15 DNA (1 nM) were added to the PURE system ((−) 10-CHO-THF) containing 5 μM DnaK, 1 μM DnaJ, 1 μM GrpE, 1 μM MAP, and 1 mM acetyl-CoA. The reaction mixture was incubated at 23 °C for 24 h. (D) Extracted ion chromatograms of the methionine-excised N-terminal peptide derived from CPR1(10aa)(Q3E)-sfGFP after trypsin digestion (SEVYFDVEASK, M = 637.3010 Da, M+1 = 637.8025 Da, M+2 = 638.3038 Da, *z* = 2). The retention time was confirmed by the peptide search results from the MS/MS spectral search. (E) Extracted ion chromatograms of the methionine-excised and acetylated N-terminal peptide derived from CPR1(10aa)(Q3E)-sfGFP after trypsin digestion (S[Ace]-EVYFDVEASK, M = 658.3063 Da, M+1 = 658.8078 Da, M+2 = 659.3091 Da, *z* = 2). The retention time was confirmed by the peptide search results from the MS/MS spectral search.

The second amino acid of the target protein is acetylated by NatA after the initial methionine, not formylmethionine, is cleaved by MAP.^1^ We purified *E. coli* MAP from overexpressed cells to introduce it into the PURE system (Supplementary Figure 5A). SDS-PAGE analysis revealed that when CPR1(10aa)(Q3E)-sfGFP was synthesized without 10-CHO-THF but with purified MAP, the synthesized CPR1(10aa)(Q3E)-sfGFP slightly shifted to a higher molecular weight (Figure 2A). MS analysis showed that the N-terminal peptide with the initial methionine almost disappeared with the addition of MAP, whereas the N-terminal peptide without the initial methionine appeared (Supplementary Figure 5B,C).

Yeast NatA consists of yNaa10 (encoded by ARD1) and yNaa15 (encoded by NAT1), a transferase and an auxiliary protein, respectively.^16^ Since yNaa10 was soluble when it was synthesized at 23 and 30 °C, whereas yNaa15 was soluble only at 23 °C (Supplementary Figure 6A), the protein synthesis reaction was performed at 23 °C in the subsequent experiment. Equal amounts of yNaa10 and yNaa15 were synthesized at 23 °C when their template DNA was added at a 1:5 ratio (Supplementary Figure 6B,C). CPR1(10aa)(Q3E)-sfGFP was synthesized with or without NatA in the presence of acetyl-CoA (Figure 2B). During SDS-PAGE analysis, the mobility of CPR1(10aa)(Q3E)-sfGFP changed slightly when yNaa10 and yNaa15 were synthesized simultaneously (Figure 2C, lane 3). In addition, the acetylated N-terminal peptide was detected through MS only in the presence of both yNaa10 and yNaa15 (Figure 2D,E and Supplementary Figure 7). These results show that NatA-mediated acetylation of nascent proteins synthesized using the PURE system requires an auxiliary protein, yNat15.^16^

### NMT-Mediated Myristoylation

Since NMT reacts to the second glycine after the methionine cleavage by MAP, which is similar to the reaction of NatA, we investigated the removal of the initial methionine of Goα(8aa)(C3S)-sfGFP synthesized in the presence of MAP. However, the efficiency of removing the initial methionine on Goα(8aa)(C3S)-sfGFP was less than that of CPR1(10aa)(Q3E)-sfGFP: ∼60% of the peptide with methionine at the N-terminus remained when Goα(8aa)(C3S)-sfGFP was synthesized (Supplementary Figure 5D–F). Since the amounts of synthesized products were almost the same between CPR1(10aa)(Q3E)-sfGFP and Goα(8aa)(C3S)-sfGFP, MAP activity could be highly dependent on the N-terminal amino acid sequence of the target protein.

**Figure 3:**
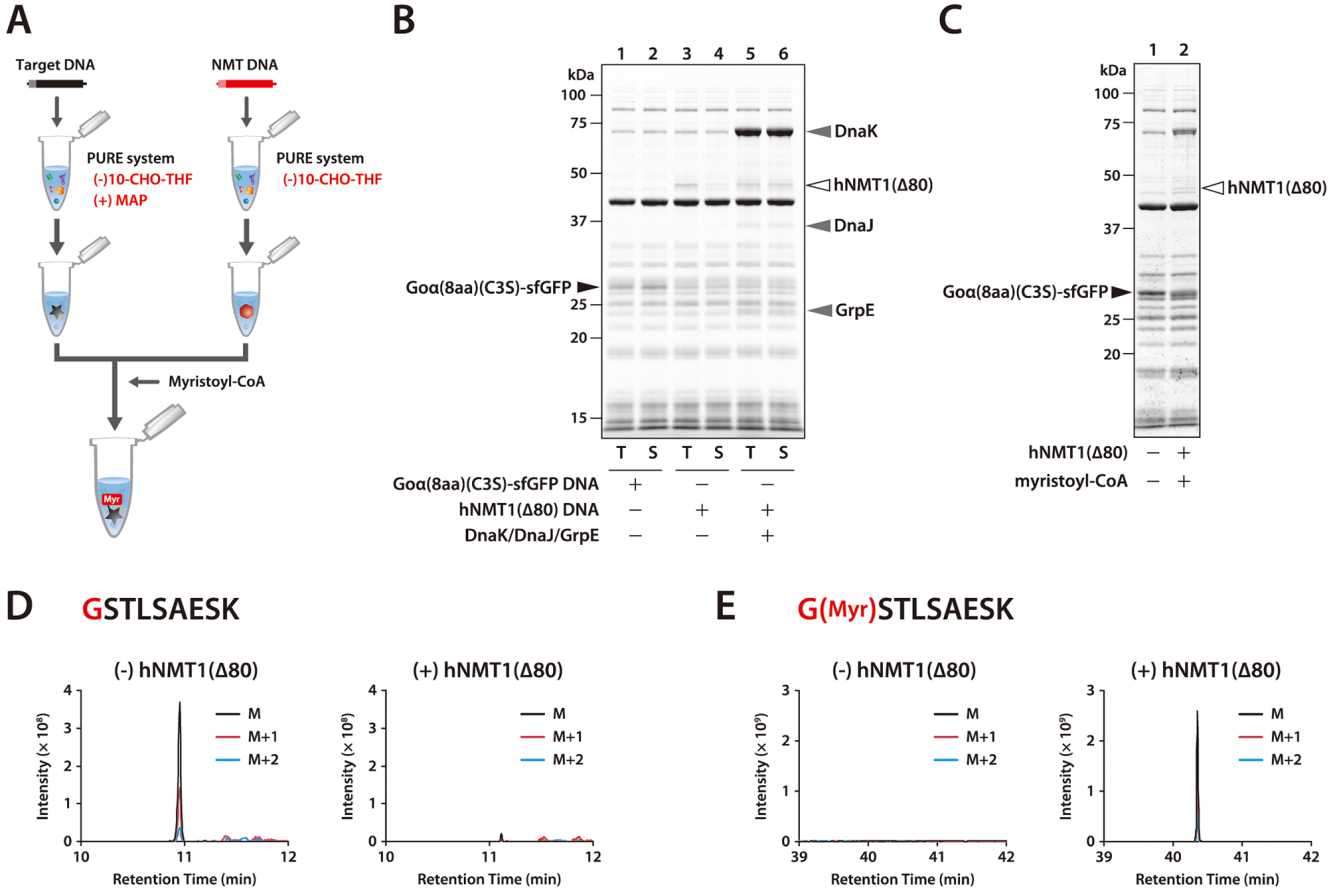
NMT-mediated post-translational N-terminal myristoylation of Goα(8aa)(C3S)-sfGFP. (A) Schematic outline of NMT-mediated post-translational N-terminal myristoylation. (B) SDS-PAGE analysis of synthesized Goα(8aa)(C3S)-sfGFP and hNMT1(Δ80). Goα(8aa)(C3S)-sfGFP was synthesized using the PURE system ((−) 10-CHO-THF) containing 1 μM MAP at 37 °C for 4 h. hNMT1(Δ80) was synthesized using the PURE system ((−) 10-CHO-THF) in the presence or absence of 5 μM DnaK, 1 μM DnaJ, and 1 μM GrpE at 30 °C for 24 h. The molecular weights of DnaK, DnaJ, and GrpE are 69, 41, and 22 kDa, respectively. The bands of the synthesized products, DnaK, DnaJ, and GrpE are indicated by arrowheads. (C) SDS-PAGE analysis of Goα(8aa)(C3S)-sfGFP incubated with hNMT1(Δ80) and myristoyl-CoA. After synthesis of Goα(8aa)(C3S)-sfGFP and hNMT1(Δ80) separately, as described in (B), the reaction mixture was mixed and incubated in the presence of 100 μM myristoyl-CoA at 30 °C for 24 h. (D) Extracted ion chromatograms of the methionine-excised N-terminal peptide derived from Goα(8aa)(C3S)-sfGFP after trypsin digestion (GSTLSAESK, M = 440.2245 Da, M+1 = 440.7260 Da, M+2 = 441.2272 Da, *z* = 2). The retention time was confirmed by the peptide search results from the MS/MS spectral search. (E) Extracted ion chromatograms of the methionine-excised and myristoylated N-terminal peptide derived from Goα(8aa)(C3S)-sfGFP after trypsin digestion (G[Myr]-STLSAESK, M = 545.3237 Da, M+1 = 545.8252 Da, M+2 = 546.3266 Da, *z* = 2). The retention time was confirmed by the peptide search results from the MS/MS spectral search.

Myristoylation and acetylation of nascent polypeptides are both cotranslational events in a cell; therefore, we first performed cosynthesis of the target protein and NMT in the same tube in the presence of myristoyl-CoA. However, we found that 50 μM myristoyl-CoA inhibited protein synthesis, whereas acetyl-CoA did not inhibit protein synthesis even at 1 mM (Supplementary Figure 8). Therefore, we synthesized the target protein and NMT separately and then mixed them with myristoyl-CoA (Figure 3A). Goα(8aa)(C3S)-sfGFP was successfully synthesized as a soluble protein at 30 °C (Figure 3B, lanes 1 and 2). In contrast, synthesized hNMT1(Δ80), which is truncated human NMT1, was insoluble at 30 °C. This insolubility was overcome by adding DnaK, DnaJ, and GrpE to the reaction mixture, resulting in the successful synthesis of soluble hNMT1(Δ80) at 30 °C (Figure 3B, lanes 5 and 6). After Goα(8aa)(C3S)-sfGFP and hNMT1(Δ80) were separately synthesized, the reaction mixture containing the products was mixed and incubated in the presence of myristoyl-CoA for 24 h (Figure 3C). The myristoylated peptide was detected only in the presence of hNMT1(Δ80) (Figure 3D,E and Supplementary Figure 9), indicating that Goα(8aa)(C3S)-sfGFP synthesized using the PURE system could be post-translationally myristoylated by hNMT1(Δ80).

### Membrane Localization of Myristoylated sfGFP

**Figure 4:**
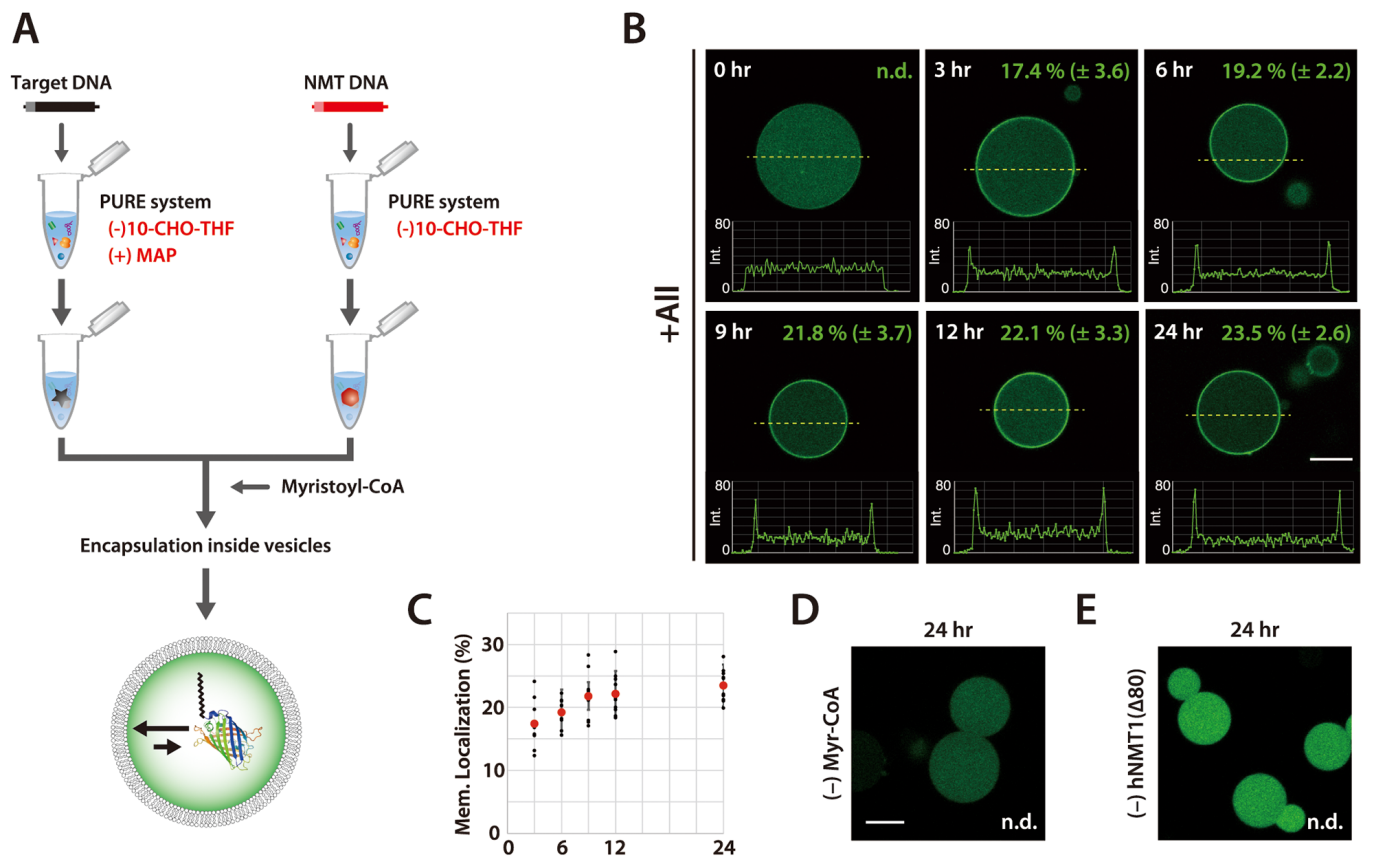
Membrane localization of myristoylated protein in giant vesicles. (A) Schematic of the experimental procedure. (B) Goα(8aa)(C3S)-sfGFP lacking the first methionine was myristoylated by hNMT1(Δ80) in giant vesicles, and the migration of the products to the vesicle membrane was observed through confocal microscopy. Wide-angle images are provided in Supplementary Figure 10. The fluorescence intensity of sfGFP was measured by the tool of plot profile in ImageJ software, along the equator of the vesicles. The ratio of membrane localization of the myristoylated Goα(8aa)(C3S)-sfGFP was calculated as described in Methods. Average and standard deviation obtained from 10 vesicles were indicated at each time. (C) The ratio of membrane localization of Goα(8aa)(C3S)-sfGFP was plotted against the reaction time at 3, 6, 9, 12, and 24 h. The localization of sfGFP was not observed when myristoyl-CoA (D) or the hNMT1(Δ80) gene (E) was omitted. Bars indicate 20 μm.

Finally, we observed the localization of myristoylated Goα(8aa)(C3S)-sfGFP to the lipid membrane in giant vesicles. The PURE system mixtures containing synthesized Goα(8aa)(C3S)-sfGFP and hNMT1(Δ80) were mixed with myristoyl-CoA and encapsulated within giant vesicles (Figure 4A). Giant vesicles formed in this manner allow the myristoylation of Goα(8aa)(C3S)-sfGFP as well as the simultaneous localization of myristoylated Goα(8aa)(C3S)-sfGFP to the vesicle membrane. We observed that myristoylated Goα(8aa)(C3S)-sfGFP localized to the vesicle membrane 3 h after the reaction was initiated (Figure 4B). The percentage of membrane-localized proteins per whole, encapsulated proteins, as estimated by plot profile measurement of the vesicles, slightly increased to 22.1 ± 3.3% (*n* = 10) within 12 h (Figure 4C). In contrast, no membrane localization was observed when myristoyl-CoA or the hNMT1(Δ80) gene was omitted from the mixture (Figure 4D,E and Supplementary Figure 10). We also investigated the effect of the membrane lipid composition. However, we did not observe a significant difference when 30 mol % cholesterol was introduced into the lipid composition (Supplementary Figure 11A) or when 1-palmitoyl-2-oleoyl-*sn*-glycero-3-phospho-(1′-*rac*-glycerol) (POPG), a negatively charged phospholipid, was eliminated (Supplementary Figure 11B). Even when molecular crowding was eliminated by removing Ficoll PM70, the membrane localization was comparable to that of + Ficoll (Supplementary Figure 11C). These results indicate that the myristoylated Goα(8aa)(C3S)-sfGFP localized to the membrane only through the force of the hydrophobic interaction between the lipid membrane and the myristoyl chain. In our study, not all of the proteins were localized to the membrane. There are two possible explanations for this observation. The first is the low myristoylation efficiency. Since the excision efficiency of the first methionine of Goα(8aa)(C3S)-sfGFP by MAP was low (Supplementary Figure 5), unmyristoylated Goα(8aa)(C3S)-sfGFP was present inside the vesicles. The second possibility is the low hydrophobicity of the myristoylated proteins. In a cell, wild-type Goα is palmitoylated (C16) after myristoylation (C14) and then localized to the membrane.^17^ However, our experimental setup does not include the palmitoylation pathway. Therefore, myristoylated Goα(8aa)(C3S)-sfGFP may be in an equilibrium state between the membrane and the vesicle lumen because the hydrophobicity of the myristoyl chain was not strong enough.

## Conclusions

In this study, we developed a preparation method for the N-terminal modification of cell-free synthesized proteins using the PURE system. As the PURE system is a reconstituted system containing only the factors necessary for protein synthesis, unmodified proteins can be easily synthesized without any changes to the mixture. Furthermore, our results showed that modified proteins were produced by simply adding the enzymes and their substrates necessary for the reaction. However, some additives might inhibit protein synthesis; thus, modification conditions should be considered in the experimental design. We also synthesized the modifying enzymes using the PURE system and used them without purification. This approach may be useful for evaluating the activity of modifying enzymes without the use of live cells. Our PURE-system-based method can be applied to achieve not only acetylation and myristoylation but also other modification systems.

## Methods

### Preparation of Template DNA for Protein Synthesis

All template DNA sequences for cell-free protein synthesis were designed using CodHonEditor^18^ based on *E. coli* codon usage and synthesized by GenScript (Supplementary Table 1). The template DNA contained the 5′-UTR (5′-GAAATTAATACGACTCACTATAGGGAGACCACAACGGTTTCCCTCTAGAAATAATTTTGTTTAACTTTAAGAAGGAGATATACCA-ORF-3′), including the T7 promoter and Shine–Dalgarno sequence, and the 3′-UTR (5′-ORF-TAATGAATAACTAATCC-3′).

### Purification of *E. coli* MAP

DNA encoding *E. coli* MAP (UniProt ID P0AE18) was amplified from *E. coli* TG1 genomic DNA and cloned into a pET15b expression vector (Novagen). *E. coli* BL21 (DE3) cells were transformed with the plasmid, and N-terminal His-tagged MAP was expressed by adding 0.1 mM IPTG. Overexpressed MAP was purified through metal affinity chromatography using Ni-Sepharose 6 Fast Flow (Cytiva) and ion exchange chromatography using Q-Sepharose Fast Flow (Cytiva) according to the manufacturer’s instructions. Purified MAP was concentrated and buffer-exchanged by dialyzing against 20 mM HEPES-KOH (pH 7.6), 200 mM potassium acetate, 7 mM 3-mercapto-1,2-propanediol, and 30% glycerol.

### Protein Synthesis and N-Terminal Modification

PURE*frex* 2.1 (GeneFrontier, Japan) was used as the PURE system reagent for cell-free protein synthesis. In most cases, the reagent without 10-CHO-THF was used (PURE system [(−) 10-CHO-THF]). DnaK mix (20× solution: 100 μM DnaK, 20 μM DnaJ, and 20 μM GrpE; GeneFrontier) was added at the indicated concentration for the synthesis of aggregated-prone proteins. Detailed reaction conditions are described in the figure legends.

### Solubility Assay of Synthesized Proteins

To confirm the solubility of the synthesized proteins, the reaction mixtures were centrifuged at 20,000*g* for 30 min after protein synthesis. Thereafter, 0.5 μL of the reaction mixture and the supernatant were subjected to reduced SDS-PAGE (12.5% or 10–20% w/v gradient). The gels were stained with SYPRO Orange protein gel stain (Thermo Fisher Scientific). Protein bands were visualized and quantitated using a WSE-6300 LuminoGraph III (ATTO, Japan) and CS analyzer 4 software (ATTO).

### Myristoylation of sfGFP Inside Giant Vesicles

Goα(8aa)(C3S)-sfGFP and hNMT1(Δ80) were synthesized as mentioned above. The reaction mixtures were centrifuged at 20,000*g* for 30 min at 4 °C, and the resulting supernatants were collected and stored at −80 °C until use. The inner solution of the vesicles was prepared by mixing 20 mM HEPES-KOH (pH 7.6), Goα(8aa)(C3S)-sfGFP reaction mixture, hNMT1(Δ80) reaction mixture, myristoyl-CoA, and sucrose (Supplementary Table 2). The resulting solution was mixed with 2.4 mg of Ficoll PM70 and completely dissolved. The outer solution was prepared as described in Supplementary Table 2. The prepared inner solution was encapsulated inside giant vesicles using the outer solution and a phospholipid-dissolving oil, as described previously.^19^ The vesicles were primarily composed of the following phospholipids: 80% 1-palmitoyl-2-oleoyl-*sn*-glycero-3-phosphocholine (POPC) (Avanti Polar Lipids) and 20% 1-palmitoyl-2-oleoyl-*sn*-glycero-3-phospho-(1′-*rac*-glycerol) (POPG) (sodium salt; Avanti Polar Lipids). The resulting vesicle mixtures were incubated at 30 °C for specific periods, as described in the figure legends.

### Microscopy Observations

Giant vesicles were observed using a Nikon A1R confocal microscopy system equipped with a 488 nm HV laser (GaAsP, 50; offset, 0; intensity, 5.0; scan size, 1024; scan speed, 0.5 frame/s; pixel dwell time:, 1.09 μs). In all cases, the images were captured as a set with differential interference contrast microscopy. The ratio of membrane localization of myristoylated Goα(8aa)(C3S)-sfGFP was estimated as follows. The fluorescence intensity of sfGFP was measured by the tool of plot profile in ImageJ software along the equator of the vesicles. Based on the obtained values, the sum of both edge intensities was divided by the total sum of intensities in the vesicle to calculate the percentage. For each sample, 10 vesicles were measured to calculate the standard deviation.

### Detection of the N-Terminal Peptides by LC-MS/MS

For MS analysis, the reaction mixtures were centrifuged at 20,000*g* for 30 min, and 20 μL of the supernatant was subjected to reduced SDS-PAGE (12.5% or 10–20% w/v gradient). Gels were stained with SimplyBlue SafeStain (Thermo Fisher Scientific). Stained bands were excised and cut into small pieces. The gel pieces were placed in a microtube and destained by washing at least twice with Solution 1 (50 mM ammonium bicarbonate and 30% acetonitrile). Thereafter, the gels were dehydrated by washing three times with Solution 2 (50 mM ammonium bicarbonate and 60% acetonitrile). The gels were evaporated by using a centrifugal evaporator. The dried gels were soaked with 40–60 μL of 50 mM ammonium bicarbonate solution containing 0.013 mg/mL Trypsin Gold (Promega, cat. no. V5280) and incubated at 37 °C overnight for digestion. After digestion, the supernatant was collected, and 200 μL of 50% acetonitrile solution was added to the remaining gels, which were shaken at 37 °C for 30 min to extract the digested peptides. After extraction, the supernatant was mixed with the supernatant collected prior to extraction. The solution was evaporated by using a centrifugal evaporator. The dried peptides were dissolved in 2% acetonitrile and 0.1% trifluoroacetic acid solution and desalted using a GL-Tip SDB (GL Sciences, Japan) according to the manufacturer’s instructions. The peptides eluted with 80% acetonitrile and 0.1% trifluoroacetic acid solution were evaporated by using a centrifugal evaporator and redissolved in 2% acetonitrile and 0.1% trifluoroacetic acid solution.

LC-MS/MS measurements were performed using an Easy-nLC1000 nanoflow liquid chromatography system and a Q-Exactive tandem mass spectrometer equipped with a nano-ESI ion source (Thermo Fisher Scientific). The trap column was a 2 cm × 75 μm capillary column packed with 3 μm C18-silica particles (Thermo Fisher Scientific), and the separation column was a 12.5 cm × 75 μm capillary column packed with 3 μm C18-silica particles (Nikkyo Technos, Japan). The flow rate was set at 300 L/min. Separation was conducted using a 10–40% linear acetonitrile gradient over 30 min in the presence of 0.1% formic acid. MS/MS data were acquired in data-dependent acquisition (DDA) mode controlled by the Xcalibur 4.0 program (Thermo Fisher Scientific). The DDA settings were as follows: the resolution was 70,000 for a full MS scan and 17,500 for a MS2 scan; the AGC target was 3.0 × 10^6^ for a full MS scan and 5.0 × 10^5^ for a MS2 scan; the maximum IT was 60 ms for both the full MS and MS2 scans; the full MS scan range was *m*/*z* 310–1500, and the top 10 signals in each full MS scan were selected for the MS2 scan. DDA measurements were performed two or three times for each sample as technical replicates.

The MS/MS data were analyzed using Proteome Discoverer 2.4 software bundled with the Sequest HT search engine (Thermo Fisher Scientific) to obtain the MS/MS spectra and peptide search parameters. Generation of the extracted ion chromatogram of the corresponding *m*/*z* and quantification were performed using Skyline software (version 22.2).^20^

## Supporting Information Available

Construct of the target proteins used in this study; effect of 10-CHO-THF on protein synthesis in the PURE system; synthesis of NatB using the PURE system; MS analysis of the N-terminal peptide of α-synuclein(K6A); MAP-mediated removal of the initial methionine of the synthesized protein; synthesis of NatA using the PURE system; MS analysis of the N-terminal peptide of CPR1(10aa)(Q3E)-sfGFP; inhibition of protein synthesis by myristoyl-CoA; MS analysis of the N-terminal peptide of Goα(8aa)(C3S)-sfGFP; membrane localization of myristoylated protein in giant vesicles; effect of lipid composition and molecular crowding; template DNA used for cell-free protein synthesis; solutions for myristoylation of sfGFP in giant vesicles (PDF)

## Abbreviations

10-CHO-THF: 10-formyl tetrahydrofolate
POPG: 1-palmitoyl-2-oleoyl-*sn*-glycero-3-phospho-(1′-*rac*-glycerol)
POPC: 1-palmitoyl-2-oleoyl-*sn*-glycero-3-phosphocholine
acetyl-CoA: acetyl-coenzyme A
DDA: data-dependent acquisition
MBP: maltose-binding protein
MS: mass spectrometry
MAP: methionine aminopeptidase
myristoyl-CoA: myristoyl-coenzyme A
NAT: N-acetyltransferase
NMT: N-myristoyltransferase
