# Supplementary material for: Regulated N-Terminal Modification of Proteins Synthesized Using a Reconstituted Cell-Free Protein Synthesis System
Source: ACS Synth Biol. 2023 Jun 16;12(7):1935–42. doi: 10.1021/acssynbio.3c00191 (PMC10367130; doi:10.1021/acssynbio.3c00191)
Supplement: Supplementary file 1 — sb3c00191_si_001.pdf [file sb3c00191_si_001.pdf]

SUPPORTING INFORMATION

Regulated N-terminal Modification of Proteins Synthesized Using a Reconstituted Cell-Free Protein Synthesis System

Rena Matsumoto[1], Tatsuya Niwa[2], Yasuhiro Shimane[3], Yutetsu Kuruma[3], Hideki Taguchi[2], Takashi Kanamori[1]*

[1] GeneFrontier Corporation, 273-1 Kashiwa, Kashiwa, Chiba 277-0005, Japan

[2] Cell Biology Center, Institute of Innovative Research, Tokyo Institute of Technology, Yokohama 226-8503, Japan

[3] Institute for Extra-cutting-edge Science and Technology Avant-garde Research (X-star), Japan Agency for Marine-Earth Science and Technology (JAMSTEC), 2-15 Natsushima-cho, Yokosuka, Kanagawa 237-0061, Japan

*To whom correspondence should be addressed. E-mail: kanamori@genefrontier.com

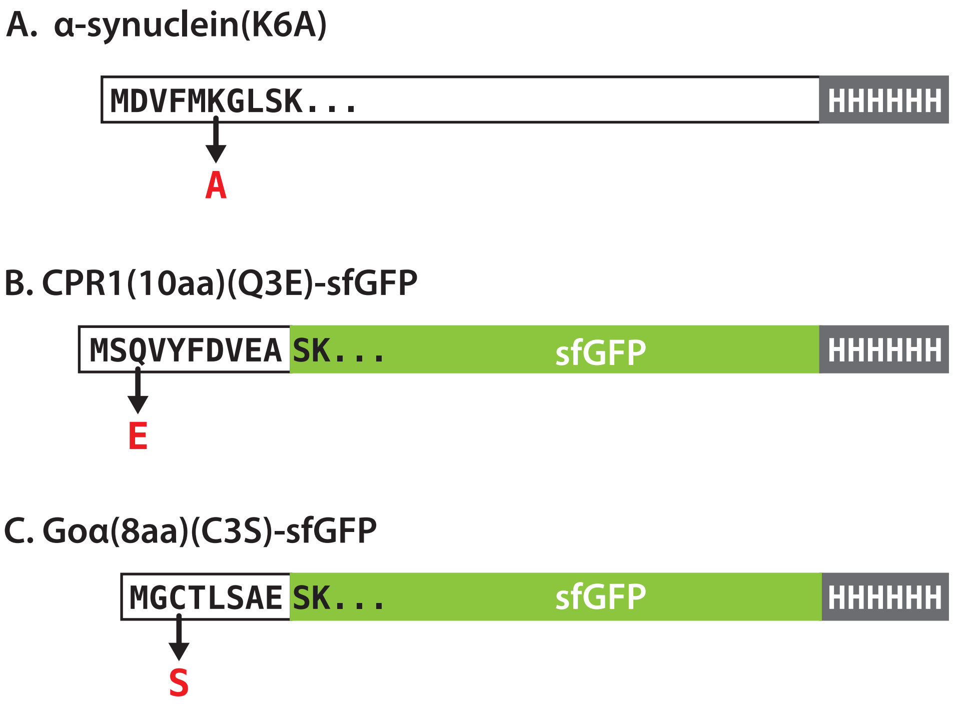

Supplementary Figure 1. The construct of the target proteins used in this study. (A) α-synuclein(K6A), (B) CPR1(10aa)(Q3E)-sfGFP, and (C) Goα(8aa)(C3S)-sfGFP. N-terminal amino acid sequence and the mutated position are shown.

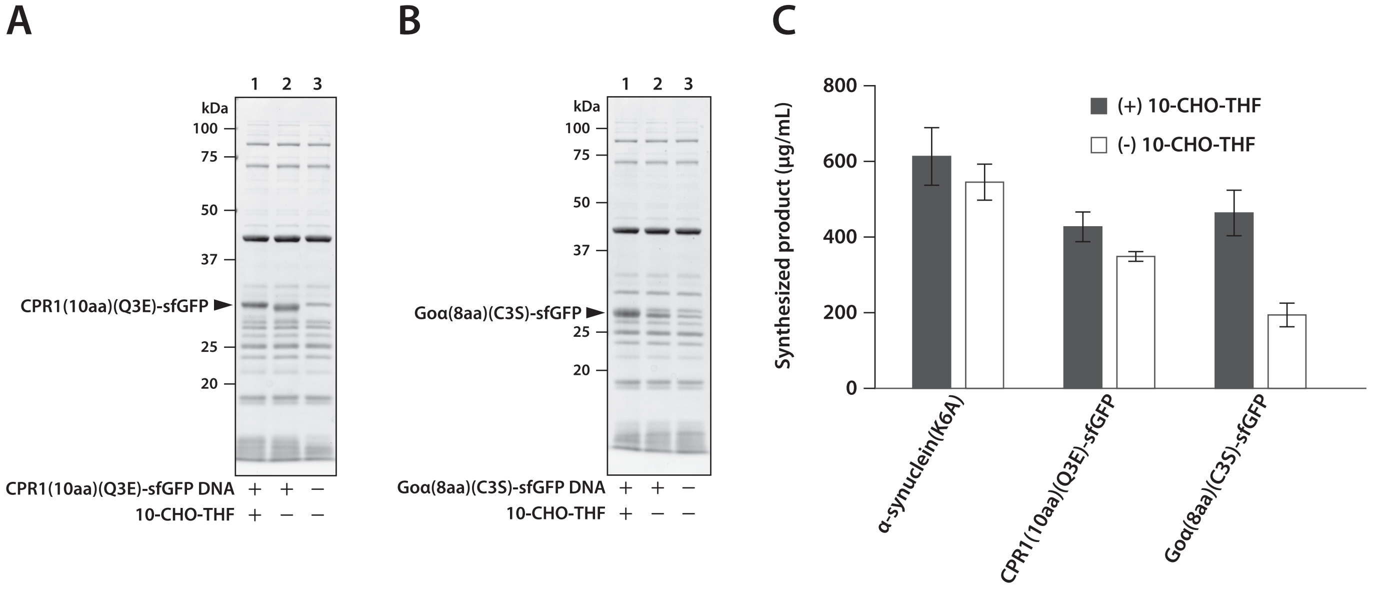

Supplementary Figure 2. Effect of 10-CHO-THF on protein synthesis in the PURE system. SDS-PAGE analysis of CPR1(10aa)(Q3E)-sfGFP (A) and Goα(8aa)(C3S)-sfGFP (B) synthesized in the presence or absence of 10-CHO-THF at 37 ^{o}C for 4 h. (C) Quantification of the synthesized α-synuclein(K6A), CPR1(10aa)(Q3E)-sfGFP, and Goα(8aa)(C3S)-sfGFP with or without 10-CHO-THF. Three experiments were performed and the average and standard deviation (SD) were calculated.

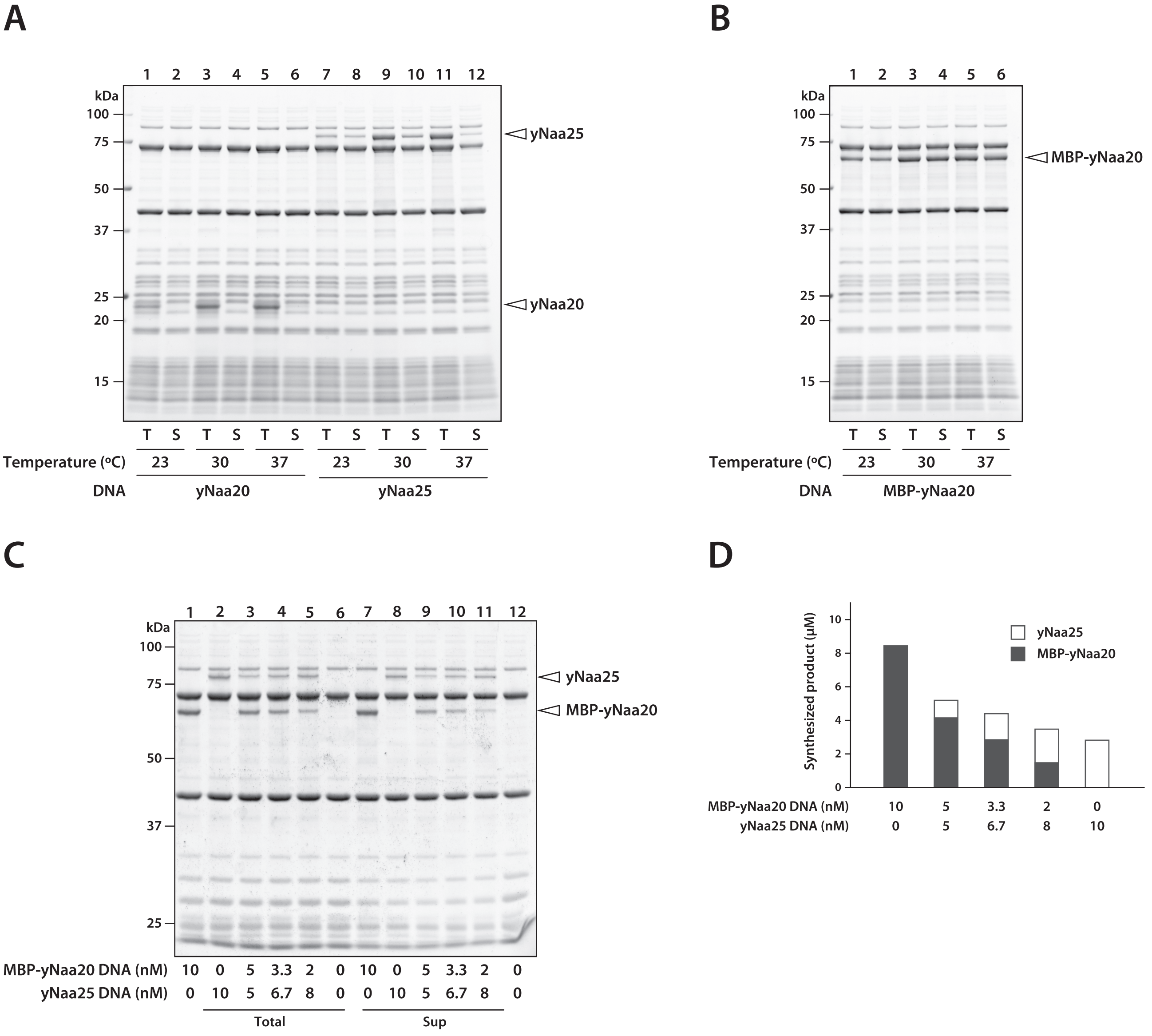

Supplementary Figure 3. Synthesis of NatB using the PURE system. SDS-PAGE analysis of synthesized NatB enzymes. yNaa20, yNaa25 (A), and MBP-yNaa20 (B) were synthesized using the PURE system ((-) 10-CHO-THF) containing DnaK, DnaJ, and GrpE at 23, 30, or 37 ºC for 4 h. After synthesis, the reaction mixture was centrifuged and the supernatant was isolated. Total reaction mixture (T) and supernatant (S) were subjected to SDS-PAGE. No soluble fractions of yNaa20 were observed even at 23 ºC. (C) MBP-yNaa20 and yNaa25 were synthesized in the same tube using the PURE system ((-) 10-CHO-THF) with the indicated ratio of the template DNA at 23 ºC for 24 h. After synthesis, the reaction mixture was centrifuged, and the supernatant was isolated. Total reaction mixture (Total) and supernatant (Sup) were subjected to SDS-PAGE. (D) Quantification of each synthesized product in (C). The synthesized amount of each product was calculated in molar concentration.

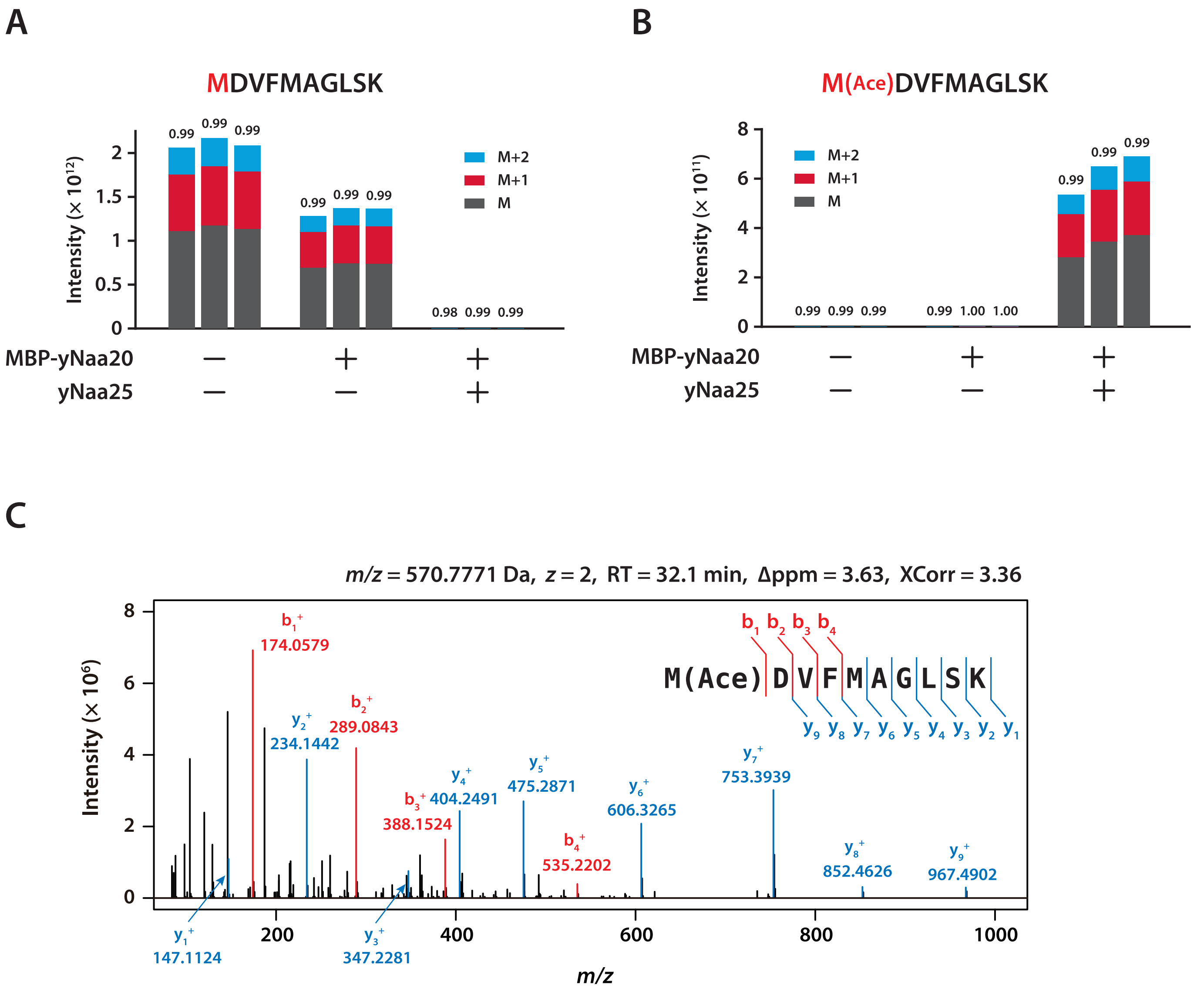

Supplementary Figure 4. MS analysis of the N-terminal peptide of α-synuclein(K6A). (A) Signal intensities of the unmodified N-terminal peptide derived from α-synuclein(K6A) after trypsin digestion (MDVFMAGLSK, M = 549.7697 Da, M+1 = 550.2712 Da, M+2 = 550.7710 Da, z = 2). The number above the bar indicates an isotope dot product (idotp) score. (B) Signal intensities of the acetylated N-terminal peptide derived from α-synuclein(K6A) after trypsin digestion (M [Ace]-DVFMAGLSK, M = 570.7750 Da, M+1 = 571.2765 Da, M+2 = 571.7764 Da, z = 2). The number above the bar indicates the idotp score. (C) MS/MS spectra of the M[Ace]-DVFMAGLSK peptide obtained from the sample when both MBP-yNaa20 and yNaa25 were synthesized simultaneously. The annotation of the fragment ions and the peptide annotation parameters were obtained from the Proteome Discoverer 2.4 software. The peaks of the b- and y- fragment ions are shown in red and blue, respectively.

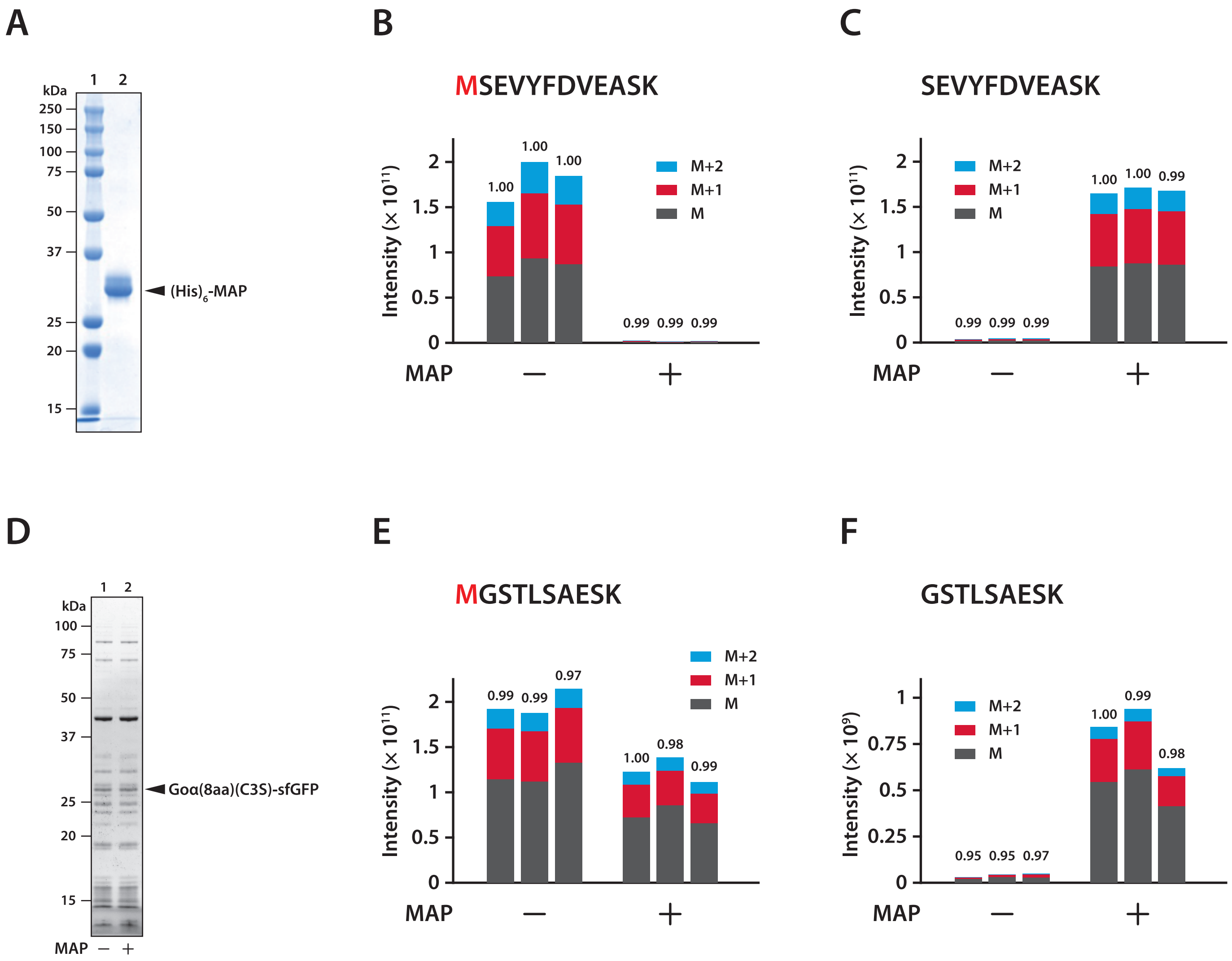

Supplementary Figure 5. MAP-mediated removal of the initial methionine of the synthesized protein. (A) SDS-PAGE analysis of purified MAP with hexahistidine tag at N-terminus. (B) Signal intensities of the N-terminal peptide derived from CPR1(10aa)(Q3E)-sfGFP after trypsin digestion (MSEVYFDVEASK, M = 702.8212 Da, M+1 = 703.3227 Da, M+2 = 703.8235 Da, z = 2). The number above the bar indicates an isotope dot product (idotp) score. (C) Signal intensities of the methionine-excised N-terminal peptide derived from CPR1(10aa)(Q3E)-sfGFP after trypsin digestion (SEVYFDVEASK, M = 637.3010 Da, M+1 = 637.8025 Da, M+2 = 638.3038 Da, z = 2). The number above the bar indicates the idotp score. (D) SDS-PAGE analysis of Goα(8aa)(C3S)-sfGFP synthesized using the PURE system ((-) 10-CHO-THF) with or without 1 μM MAP. (E) Signal intensities of the N-terminal peptide derived from Goα(8aa)(C3S)-sfGFP after trypsin digestion (MGSTLSAESK, M = 505.7448 Da, M+1 = 506.2462 Da, M+2 = 506.7464 Da, z = 2). The number above the bar indicates the idotp score. (F) Signal intensities of the methionine-excised N-terminal peptide derived from Goα(8aa)(C3S)-sfGFP after trypsin digestion (GSTLSAESK, M = 440.2245 Da, M+1 = 440.7260 Da, M+2 = 441.2272 Da, z = 2). The number above the bar indicates the idotp score.

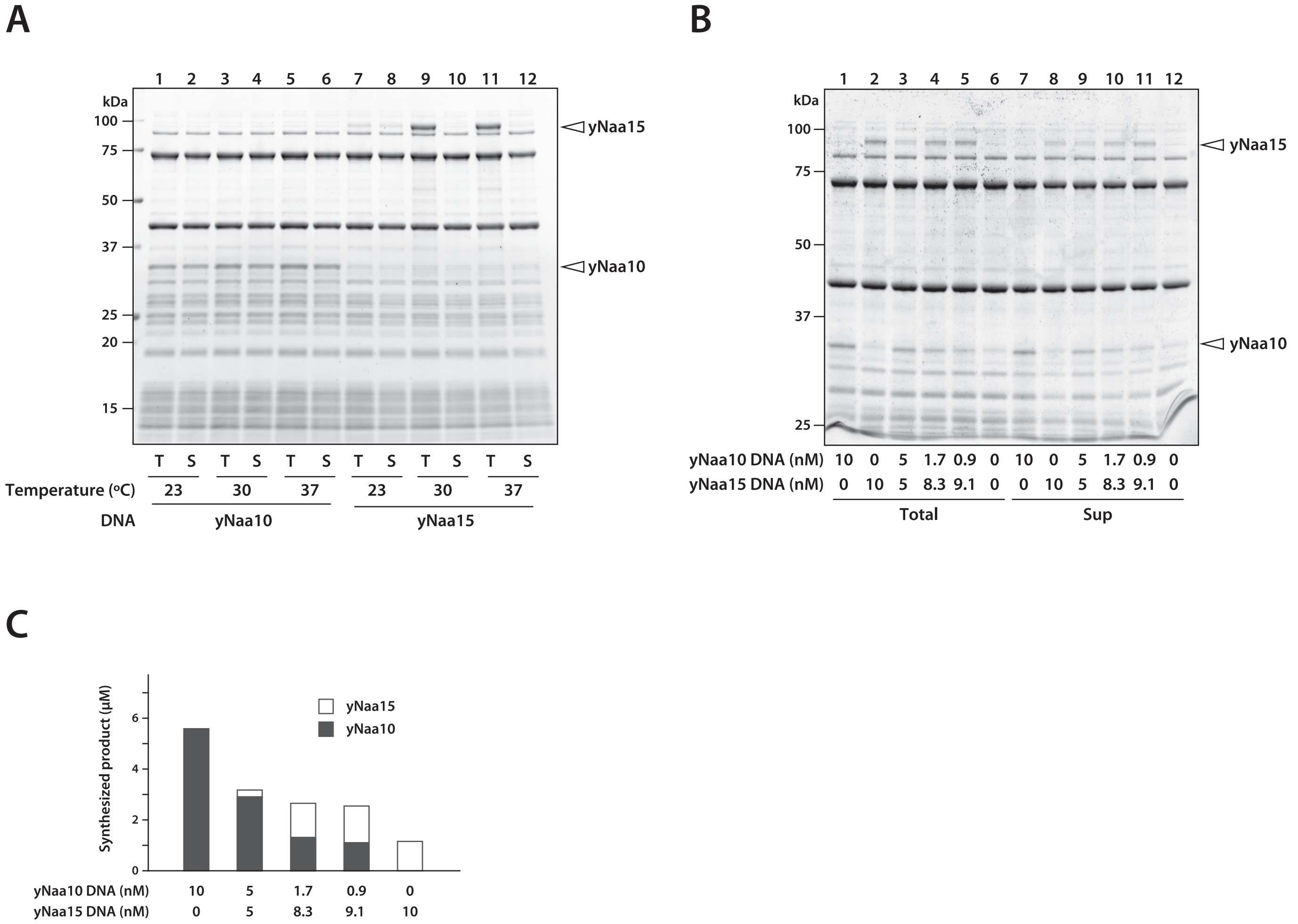

Supplementary Figure 6. Synthesis of NatA using the PURE system. (A) SDS-PAGE analysis of synthesized NatA enzymes. yNaa10 and yNaa15 were synthesized using the PURE system ((-) 10-CHO-THF) containing DnaK, DnaJ, and GrpE at 23, 30, or 37 ºC for 24 h. After synthesis, the reaction mixture was centrifuged and the supernatant was isolated. Total reaction mixture (T) and supernatant (S) were subjected to SDS-PAGE. (B) yNaa10 and yNaa15 were synthesized in the same tube using the PURE system ((-) 10-CHO-THF) with the indicated ratio of the template DNA at 23 ºC for 24 h. After synthesis, the reaction mixture was centrifuged, and the supernatant was isolated. Total reaction mixture (Total) and supernatant (Sup) were subjected to SDS-PAGE. (C) Quantification of each synthesized product in (B). The synthesized amount of each product was calculated in molar concentration.

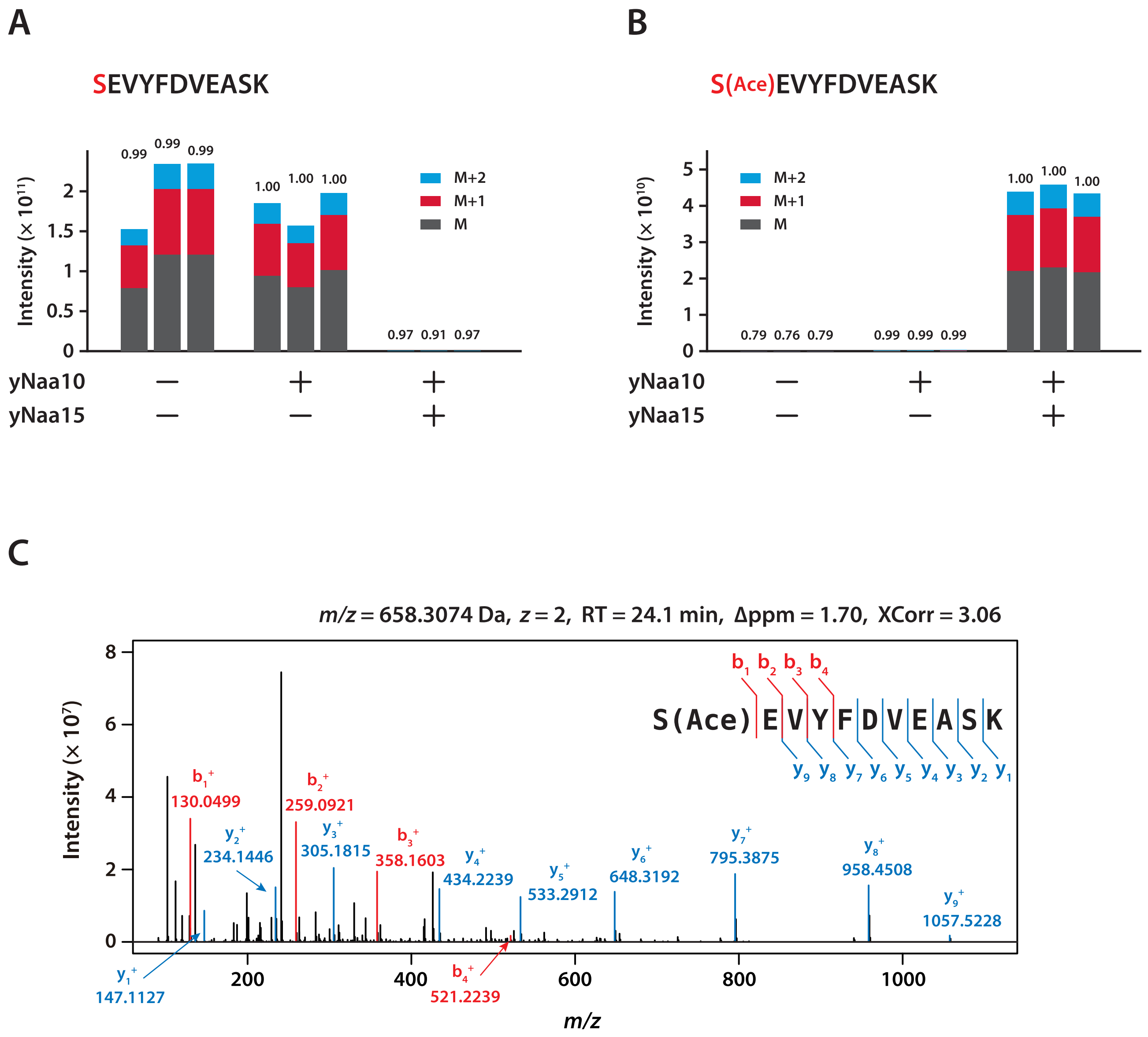

Supplementary Figure 7. MS analysis of the N-terminal peptide of CPR1(10aa)(Q3E)-sfGFP. (A) Signal intensities of the methionine-removed N-terminal peptide derived from CPR1(10aa)(Q3E)-sfGFP after trypsin digestion (SEVYFDVEASK, M = 637.3010 Da, M+1 = 637.8025 Da, M+2 = 638.3038 Da, z = 2). The number above the bar indicates an isotope dot product (idotp) score. (B) Signal intensities of the methionine-removed and acetylated N-terminal peptide derived from CPR1(10aa) (Q3E)-sfGFP after trypsin digestion (S[Ace]-EVYFDVEASK, M = 658.3063 Da, M+1 = 658.8078 Da, M+2 = 659.3091 Da, z = 2). The number above the bar indicates the idotp score. (C) MS/MS spectra of the S(Ace)-EVYFDVEASK peptide obtained from the sample when both yNaa10 and yNaa15 were synthesized simultaneously. The peaks of the b- and y- fragment ions are shown in red and blue, respectively.

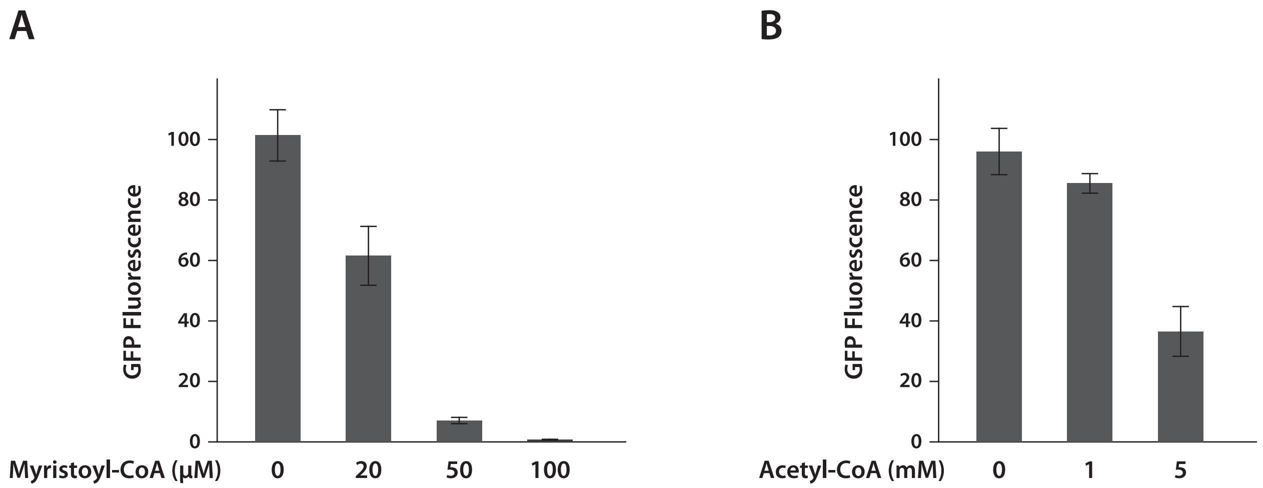

Supplementary Figure 8. Inhibition of protein synthesis by myristoyl-CoA. sfGFP was synthesized in the presence of myristoyl-CoA (A) or acetyl-CoA at the indicated concentrations. (B) The fluorescence of the synthesized sfGFP was measured. Three independent experiments were performed, and the average and standard deviations (SD) were calculated. The values are shown relative to the fluorescence at 0 µM as 100%.

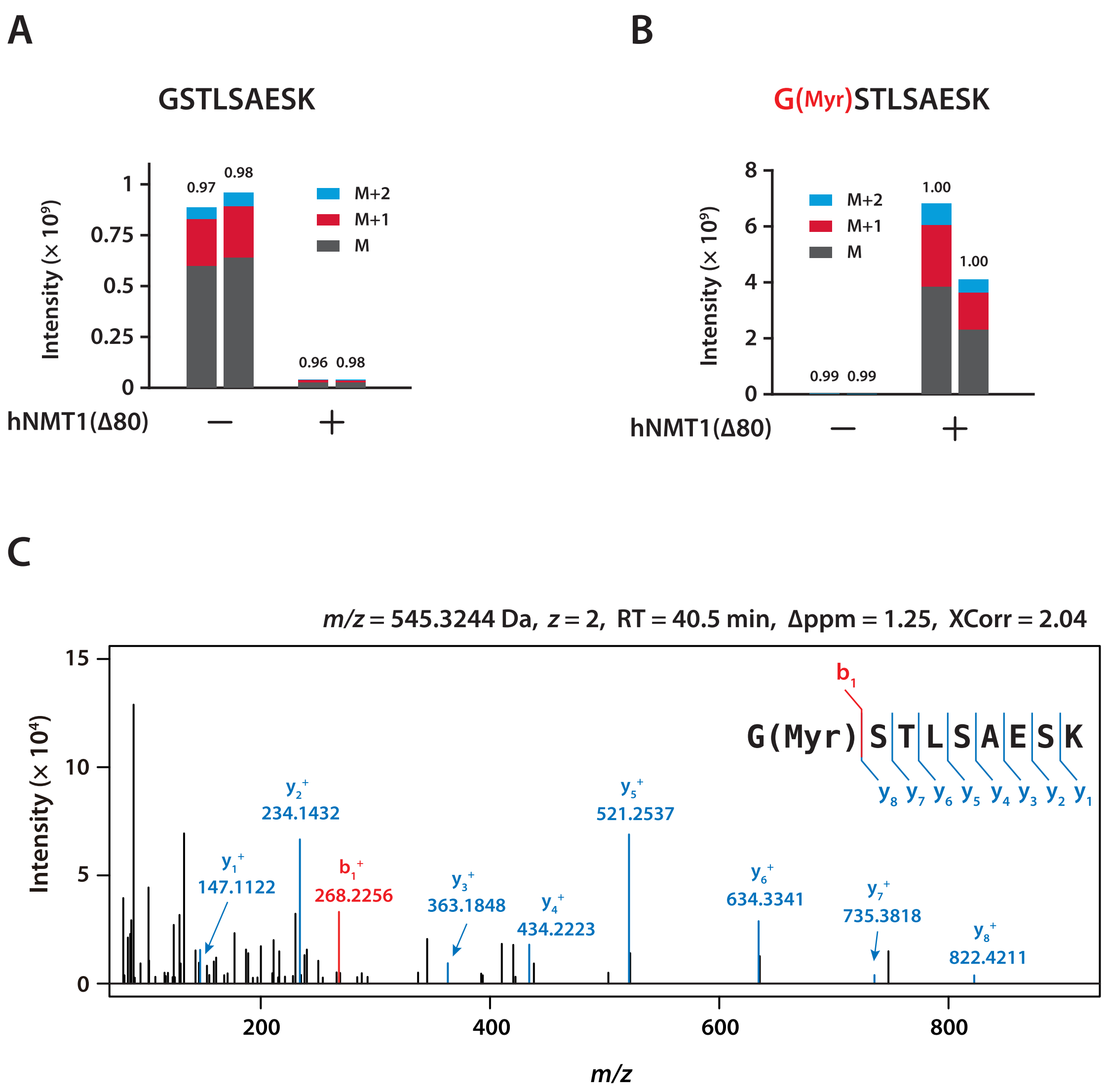

Supplementary Figure 9. MS analysis of the N-terminal peptide of Goα(8aa)(C3S)-sfGFP. (A) Signal intensities of the methionine-removed N-terminal peptide derived from Goα(8aa)(C3S)-sfGFP after trypsin digestion (GSTLSAESK, M = 440.2245 Da, M+1 = 440.7260 Da, M+2 = 441.2272 Da, z = 2). The number above the bar indicates an isotope dot product (idotp) score. (B) Signal intensities of the methionine-removed and myristoylated N-terminal peptide derived from Goα (8aa)(C3S)-sfGFP after trypsin digestion (G[Myr]-STLSAESK, M = 545.3237 Da, M+1 = 545.8252 Da, M+2 = 546.3266 Da, z = 2). The number above the bar indicates the idotp score. (C) MS/MS spectra of the G[Myr]-STLSAESK peptide obtained from the sample in the presence of hNMT1(Δ80). The annotation of the fragment ions and the peptide annotation parameters were obtained using the Proteome Discoverer 2.4 software. The peaks of the b- and y- fragment ions are shown in red and blue, respectively.

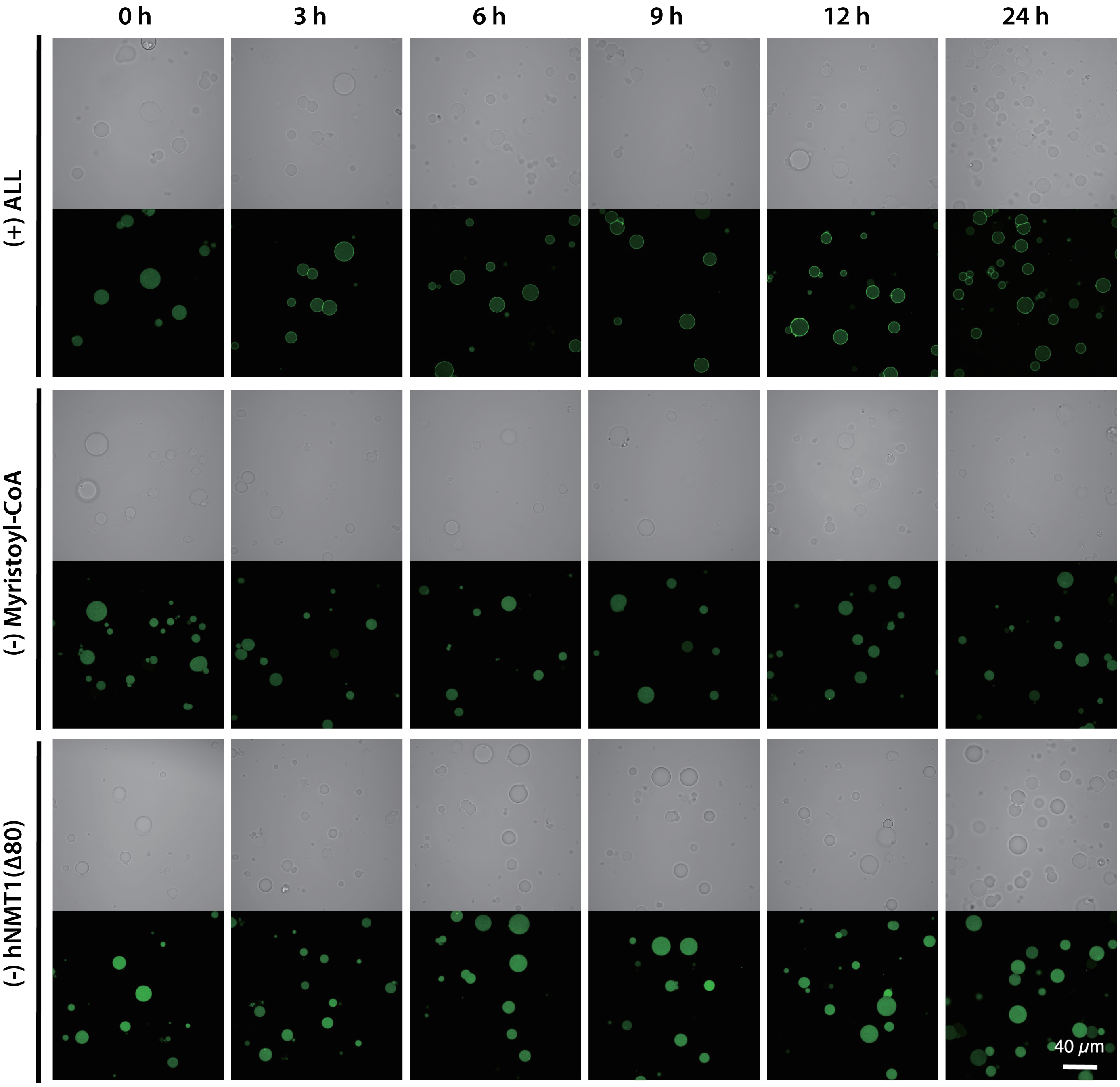

Supplementary Figure 10. Membrane localization of myristoylated protein in giant vesicles. Goα(8aa)(C3S)-sfGFP lacking methionine at the N-terminal was myristoylated by hNMT1 in giant vesicles. Migration of the products to the vesicle membrane was observed through confocal microscopy at 0, 3, 6, 9, 12, and 24 h after the reaction was initiated (top panel), with the control lacking myristoyl-CoA (middle panel) or the hNMT1(Δ80) gene (bottom panel). Bars indicate 40 μm.

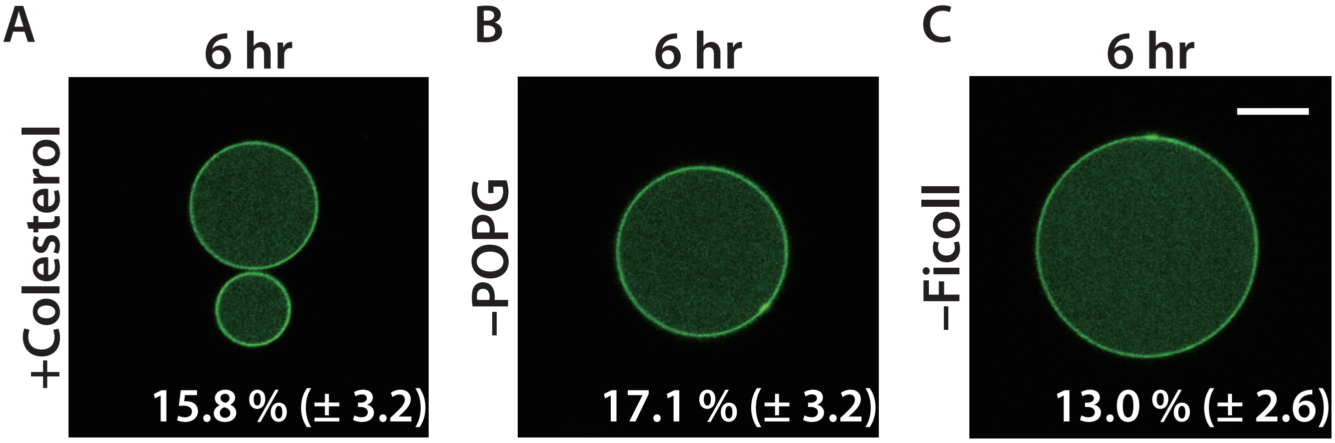

Supplementary Figure 11. The effect of lipid composition and molecular crowding. Membrane localization of myristoylated Goα(8aa)(C3S)-sfGFP was observed after 30 mol% cholesterol was added to the vesicle membrane (A) or 1-palmitoyl-2-oleoyl-sn-glycero-3-phos’ ho-(1'-rac-glycerol) (POPG) was removed from the membrane (B). Localization was also observed in the absence of Ficoll PM70 (C). Bars indicate 20 μm.

Supplementary Table 1. Template DNA used for cell-free protein synthesis.

Name	Description	Uniprot ID
yNaa20	*S. cerevisiae* Naa20 (NAT3)	Q06504
yNaa25	*S. cerevisiae* Naa25 (MDM20)	Q12387
MBP-yNaa20	yNaa20 fused with maltose-binding protein (MBP)	
yNaa10	*S. cerevisiae* Naa10 (ARD1)	P07347
yNaa15	*S. cerevisiae* Naa15 (NAT1)	P12945
hNMT1(Δ80)	*H. sapiens* NMT1 with deletion of residues 2–80	P30419
α-Synuclein(K6A)	*H. sapiens* α-Synuclein with a mutation at 6th amino acid and Histidine-tag at C-terminus	P37840 (α-Synuclein)
CPR1(10aa)(Q3E)-sfGFP	sfGFP fused with N-terminal mutated 10 amino acids (MSEVYFDVEA) of *S. cerevisiae* CPR1 and C-terminal Histidine-tag	P14832 (CPR1)
Goα(8aa)(C3S)-sfGFP	sfGFP fused with N-terminal mutated 8 amino acids (MGSTLSAE) of *H. sapiens* Goα and C-terminal Histidine-tag	P09471 (Goα)

Supplementary Table 2. Solutions for myristoylation of sfGFP in giant vesicles.

Inner solution

20 mM HEPES-KOH (pH 7.6)	5 μL	
hNMT1(Δ80) reaction mixture	6 μL	
Goα(8aa)(C3S)-sfGFP reaction mixture	6 μL	
Myristoyl-CoA§ (2 mM)	1 μL	Final concentration 100 μM
Sucrose§ (2 M)	2 μL	Final concentration 200 mM
Total	20 μL	
+ Ficoll PM70	2.4 mg	

§Dissolved with MilliQ water.

Outer solution

HEPES-KOH (pH 7.6)	20 mM
Potassium glutamate	180 mM
Magnesium acetate	14 mM
Spermidine	2 mM
Dithiothreitol	2 mM
20 amino acid mixture	0.5 mM each
Creatine phosphate	20 mM
Glucose	200 mM
